# Supplementary material for: fMRI Evidence for Default Mode Network Deactivation Associated with Rapid Eye Movements in Sleep
Source: Brain Sci. 2021 Nov 18;11(11):1528. doi: 10.3390/brainsci11111528 (PMC8615877; doi:10.3390/brainsci11111528)
Supplement: Supplementary file 1 [file brainsci-11-01528-s001.zip › brainsci-1424028-supplementary.pdf]

Supplementary Materials:

VIDEO S1

video-recording of REMs of a neonate.mp4
md5:55f458933018cc32bd0340dc46daebd8
from Hong et al (2018) with the permission from Hong.
https://drive.google.com/file/d/0BzWLE2tIe0XhYXlBRnNRRHYtM2c/view?usp=sharing.

VIDEO S2

video-recording of REMs during our fMRI study.avi
md5:e36e8957a0d523ba916adffcf51f4e71
from Hong et al (2009) with the permission from Hong
https://drive.google.com/file/d/0B0R_1dukpEw7QXpObTNZLWp0M3M/view?usp=sharing.

The following are available online at www.mdpi.com/article/ , **Figure S1:** Setup for video-recording of REMs. **Figure S2:** Subject and study characteristics associated with REM-locked signal decrease in PCu/PCC and RSC-Lt. There were no significant effects on PCu/PCC signal decrease for gender (p = .21, Fisher exact test), age (M = 24.1, SD = 3.4 for absent; M = 24.8, SD = 4.5 for present), REM sleep duration (M = 18.4 min, SD = 6.9 for absent; M = 18.2 min, SD = 8.4 for present), REM count (M = 224.2, SD = 92.1 for absent; M = 177.5, SD = 121.0 for present), and REM density per min (M = 12.3, SD = 3.2 for absent; M = 9.5, SD = 3.6 for present). There were no significant effects on RSC-Lt signal decrease for age (M = 24.8, SD = 3.6 for absent; M = 23.4, SD = 5.0 for present), REM count (M = 196.1, SD = 117.1 for absent; M = 215.0, SD = 81.0 for present), and REM density (M = 11.4, SD = 3.2 for absent; M = 9.1, SD = 4.5 for present). REM-locked signal decrease RSC-Lt occurred more often in male studies ($p < 0.05$, Fisher exact test) and is associated with longer REM sleep duration (M = 16.1, SD = 6.6 for absent; M = 25.1, SD = 6.1 for present; $t(22) = 2.9$, p = .008) (✳indicates these two statistically significant differences), However, note that for REM-locked signal decrease in RSC-Lt n is small (n = 6) and that because four of these six studies were contributed by the same subject (subject number 5, indicated by arrows), characteristics of this particular subject can skew the associative pattern. Averages and standard deviations are shown. **Figure S3:** DMN inhibitory circuit in oculomotor control.

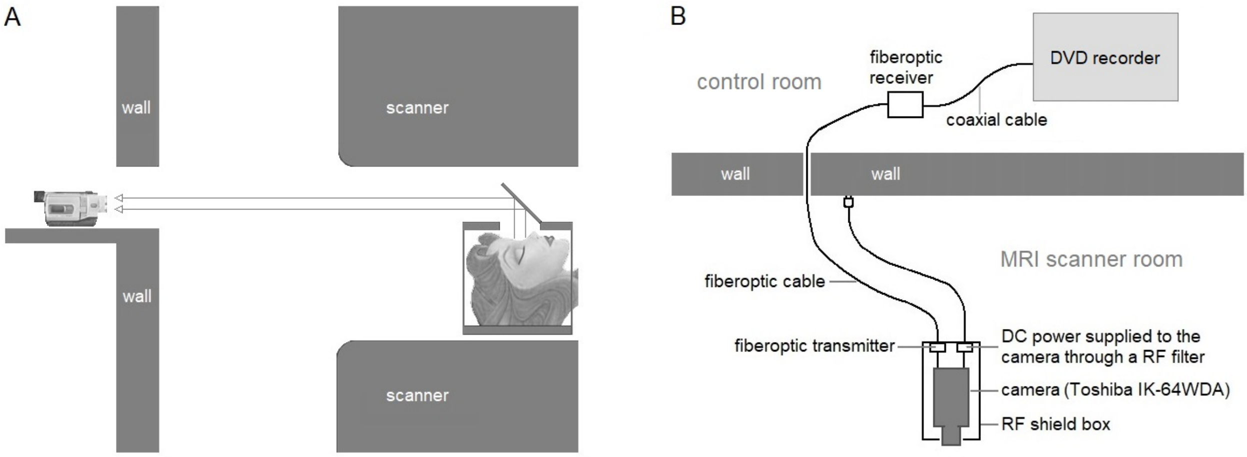

Figure S1: Setup for video-recording of REMs

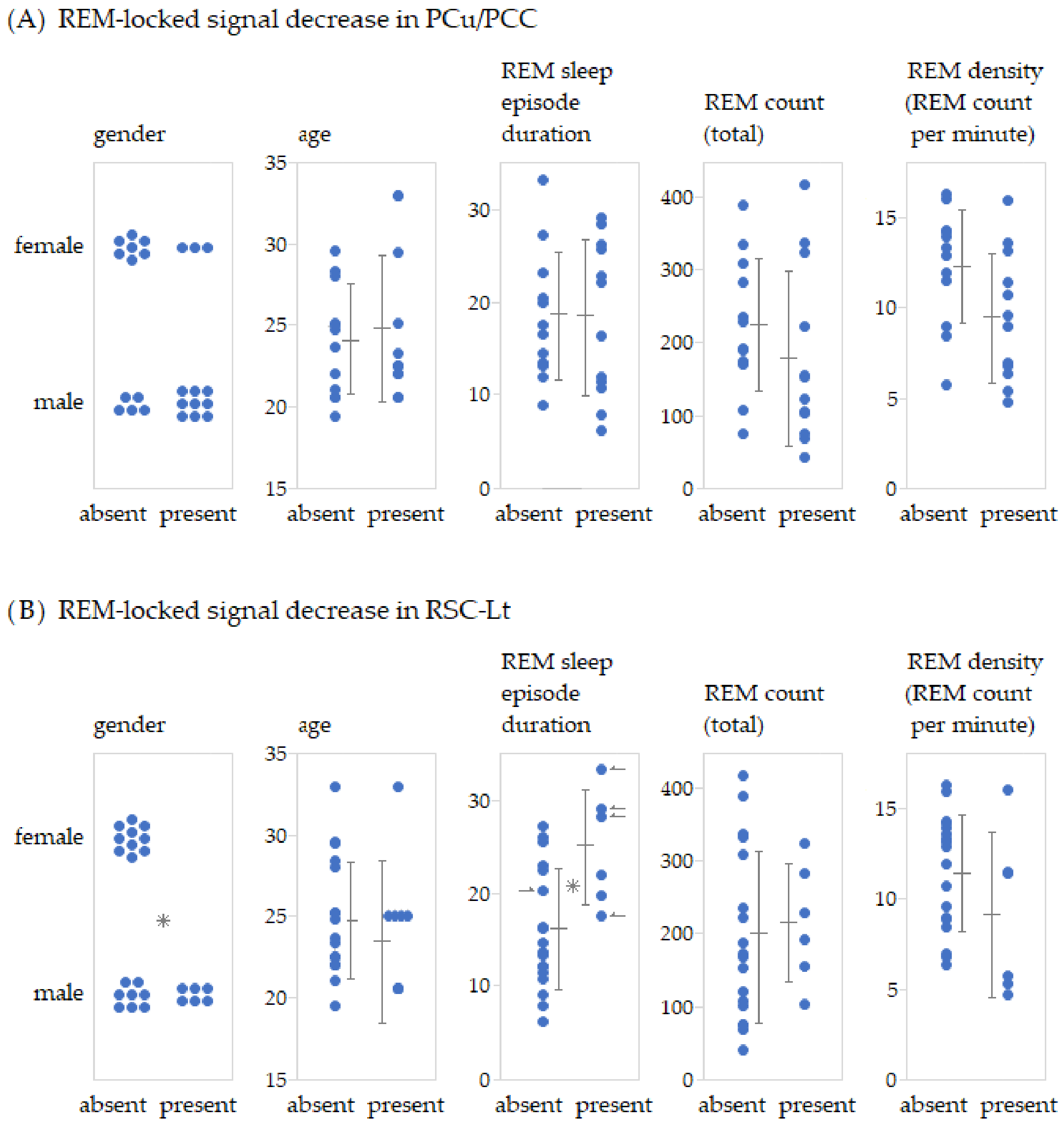

Figure S2: Subject and study characteristics associated with REM-locked signal decrease in PCu/PCC and RSC-Lt.

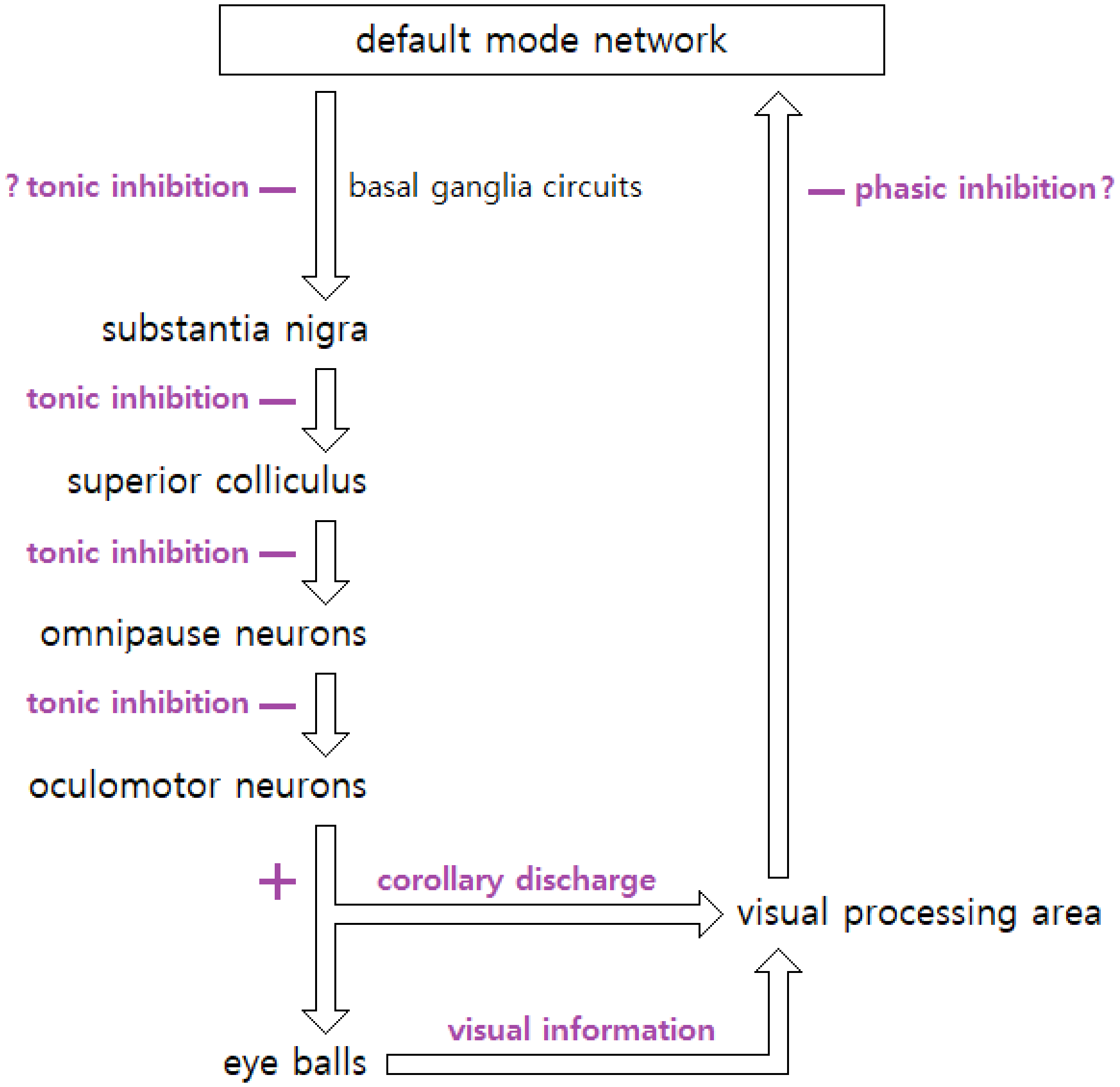

Figure S3: DMN inhibitory circuit in oculomotor control
